# Supplementary material for: α-Lack-SPI Alleviates MASLD in Rats via Regulating Hepatic Lipid Accumulation and Inflammation
Source: Nutrients. 2025 Sep 10;17(18):2918. doi: 10.3390/nu17182918 (PMC12473108; doi:10.3390/nu17182918)
Supplement: Supplementary file 1 [file nutrients-17-02918-s001.zip › nutrients-3842593-supplementary.pdf]

Supplementary Information for

α-lack-SPI alleviates MASLD in rats via regulating hepatic lipid accumulation and inflammation

Methods

Analysis of the soy protein isolate subunit composition.

The subunit composition of WT-SPI and α-lack-SPI was analyzed using sodium dodecyl sulfate–polyacrylamide gel electrophoresis (SDS-PAGE). The proteins were extracted from SPIs using SDS-PAGE loading buffer (P0285, Beyotime) and vortexed. Each sample was heated for 5 min at 100 °C, and subsequently loaded onto SDS-PAGE gels. 30/40 micrograms of each supernatant were separated on 5% stacking and 12% separating polyacrylamide gels. Next, gels were stained with Coomassie Brilliant Blue G250 solution for 1 hour, destained by washing with double-distilled water until the background was clear, and then imaged using a BIO-RAD Gel Doc XR+ system (Bio-Rad Laboratories, Hercules, CA, USA).

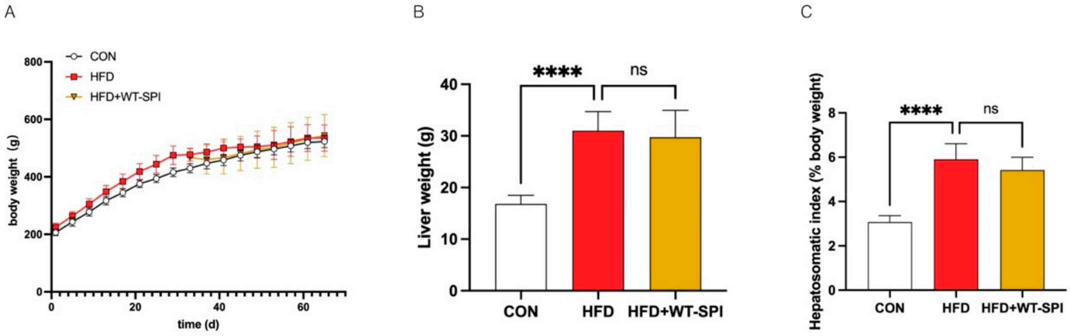

Fig S1. Effects of WT-SPI treatment on basic physiological parameters of rats with HFD-induced MASLD. (A) body weight, (B) liver weight, (C) hepatosomatic index. The data are presented as means ± SD (n=8). Compared with the HFD group, ****$p<0.0001$.

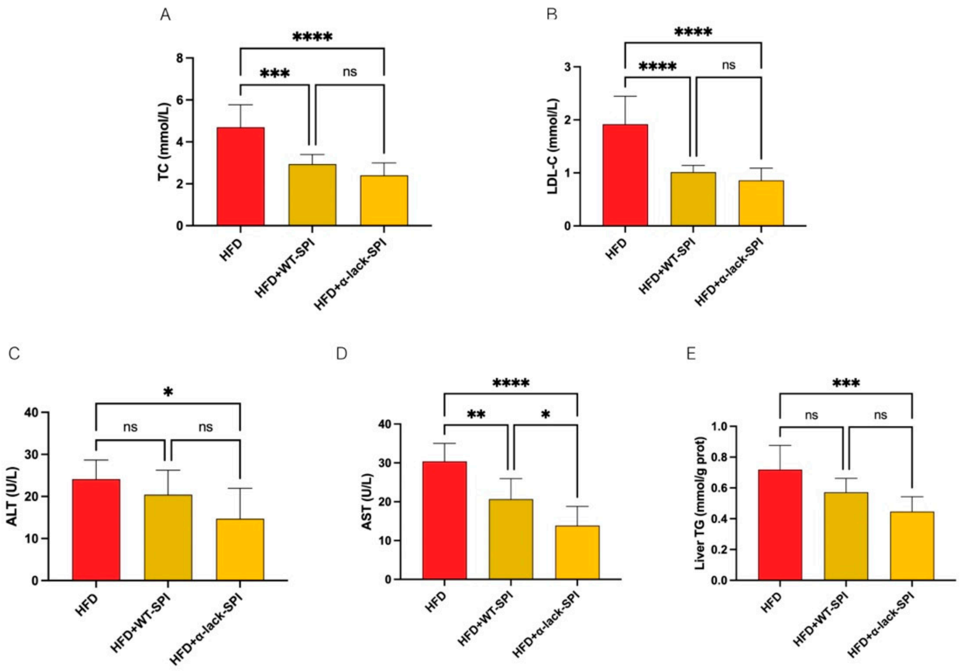

Fig S2. Effects of WT-SPI and α-lack-SPI on serum (A) TC, (B) LDL-C, (C) ALT, (D) AST, and (E) liver TG levels of rats with HFD-induced MASLD. The data are presented as means ± SD (n=8). Compared with the HFD group, *p<0.05, **p<0.01, ***p<0.001, ****p<0.0001.

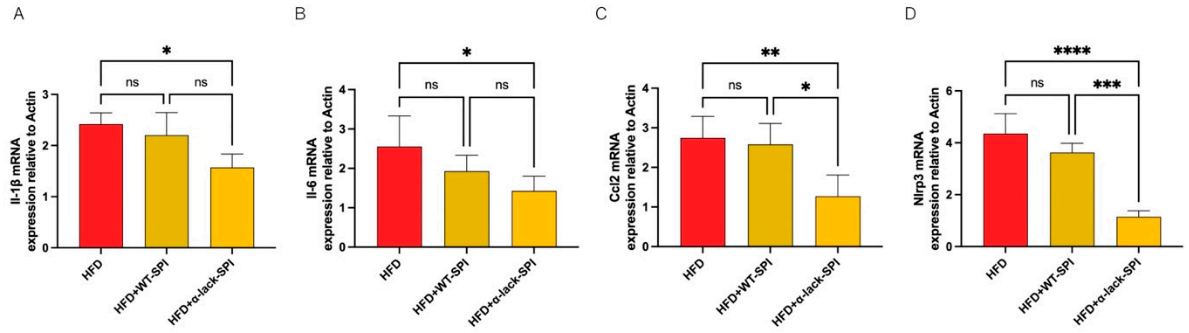

Fig S3. Effect of WT-SPI and α-lack-SPI on hepatic inflammatory mRNA and protein expressions in rats with HFD-induced MASLD. (A–D) Hepatic mRNA levels of Il-1β, Il-6, Ccl2 and Nlrp3. The data are presented as means ± SD (n=4). Compared with the HFD group, *p<0.05, **p<0.01, ***p<0.001, ****p<0.0001.

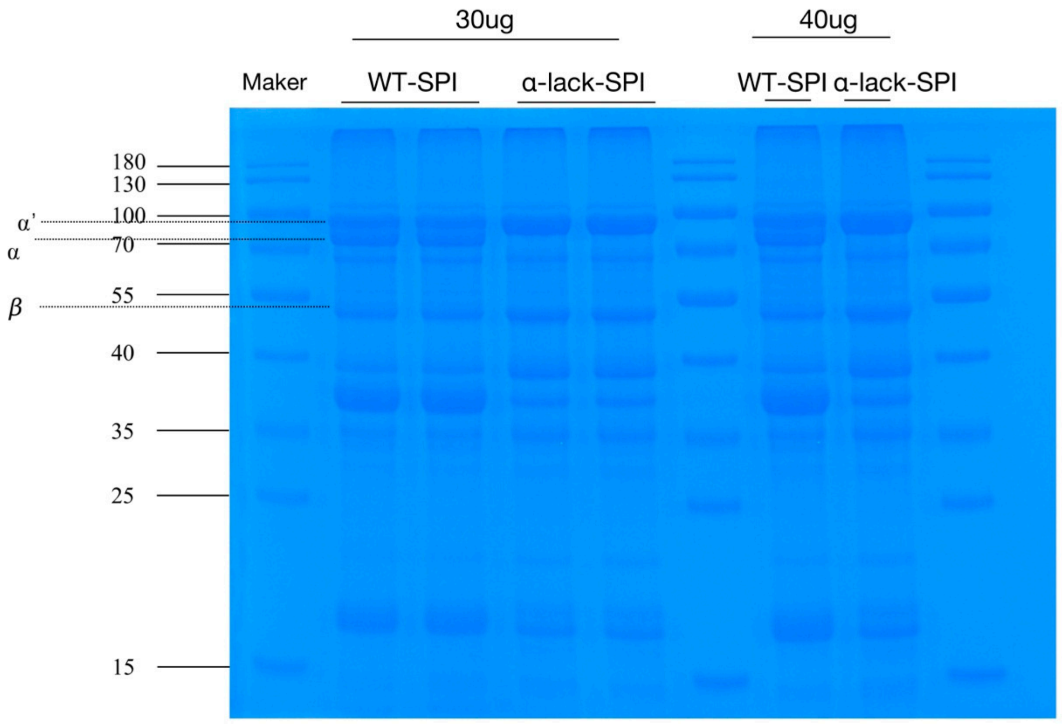

Fig S4. SDS-PAGE electrophoresis analysis of WT-SPI and α-lack-SPI.

Table S1. High fat, high cholesterol diet

Ingredients	gm	kcal
Casein	100	400
Sucrose	200	800
Lard	150	1350
Basic feed	522	1879
Premixed feed	4	0
Cholesterol	12	0
Sodium cholate	2	0
Calcium hydrogen phosphate	6	0
Calcium carbonate	4	0
Total	1000	4429
